# Life History Differences Between *Lepidoptera* Larvae and *Blattodea* Nymphs Lead to Different Energy Allocation Strategies and Cellular Qualities

**DOI:** 10.3390/insects15120991

**Published:** 2024-12-13

**Authors:** Fahimeh Taheri, Chen Hou

**Affiliations:** Department of Biology, Missouri University of Science and Technology, Rolla, MO 65409, USA; fahime.taheri@mst.edu

**Keywords:** life history, energy, growth, tradeoff, protein quality control, resistance to stress

## Abstract

Different life histories result in different strategies to allocate energy in biosynthesis, including growth and reproduction, and somatic maintenance. One of the most notable life history differences between *Lepidoptera* and *Blattodea* species is that the former grow much faster than the latter, and during metamorphosis, a large amount of tissue in *Lepidoptera* species disintegrates. In this review, using *Lepidoptera* caterpillars and cockroach nymphs as examples, we show that, due to these differences in growth processes, cockroach nymphs spend 20 times more energy on synthesizing one unit of biomass (indirect cost of growth) than butterfly caterpillars. Because of the low indirect cost of growth in caterpillars, the fraction of metabolic energy allocated to growth is six times lower, and that for maintenance is seven times higher in caterpillars, compared to cockroach nymphs, despite caterpillar’s higher growth rates. Moreover, due to the higher biosynthetic energy cost in cockroach nymphs, they have better cellular qualities, including higher proteasomal activity for protein quality control and higher resistance to oxidative stress. We also show that under food restriction conditions, the fraction of assimilated energy allocated to growth was reduced by 120% in cockroach nymphs, as they lost body weight under food restriction, while this reduction was only 14% in hornworms, and the body mass increased at a lower rate. Finaly, we discuss future research, especially the difference in adult lifespans associated with the energetic differences.

## Simple Summary

*Lepidoptera* species have higher growth rates than *Blattodea* species. The different growth rates lead to different strategies to allocate energy to biosynthesis and somatic maintenance under free-feeding and low-food-availability conditions. Moreover, *Lepidoptera* and *Blattodea* spend sharply different amounts of energy on synthesizing one unit of bio-tissue, which, in turn, leads to different cellular qualities and abilities to resist stress, and may have effect on their adult lifespan. Based on this evidence, we postulate that the capability of maintaining homeostasis not only depends on the amount of energy allocated to maintenance, but also depends on the quality of the tissue, and that the tissue quality is at least partially due to the energetic investments in biosynthesis. In short, materials that are cheap to synthesize deteriorate faster, and allocating more energy to biosynthesis enhances somatic maintenance.

## 1. Introduction

*Lepidoptera* and *Blattodea* species have sharp differences in life history. One of the most notable differences is in their developmental processes. For example, butterfly caterpillars and hornworms finish the larval development in a short period of ~2 or 3 weeks [1,2,3], but cockroach nymph’s growth period may last several months to one year [4]. Moreover, the larvae of many *Lepidoptera* species need to reach a critical weight for successful pupation [5,6], and insects’ fecundity is positively correlated to body size [7]. To reach a desired body weight in a short and limited period demands fast growth. The short growth periods and fast growth rates of moth and butterfly caterpillars result in a series of physiological features that are different than cockroach nymphs, including the energy allocation to somatic maintenance and growth under free feeding and low food availability, the energetic cost of biosynthesis, and the cellular resistance to oxidative stress, which potentially leads to the difference in adult lifespan. Here, we review a few recent developments that highlight these physiological differences between *Lepidoptera* and *Blattodea* associated with their different life histories.

## 2. Difference in Energy Allocation Strategies

### 2.1. Energy Budgets Under Free-Feeding Conditions

Different life histories result in different strategies for allocating energy in biosynthesis including growth and reproduction, and somatic maintenance, which is related to intrinsic aging [8,9,10]. It has been widely proposed [11,12] that during growth, the metabolic energy can be partitioned between the energy invested in biosynthesis (growth) and somatic maintenance as

(1) $$B=E_{m}G+B_{M,A}$$

where *B* and *B*_M,A_ are the metabolic rate and the rate of energy allocated to maintenance and activity, respectively, both in unit of energy/time; *G* is the growth rate (biomass gain/time); and *E_m_* is the metabolic cost of growth (energy/biomass). *B*, *G*, and *B*_M,A_ are all functions of age during growth. Using this equation, one can estimate the fraction of metabolic energy allocated to biosynthesis and somatic maintenance, i.e., $E_{m}G/B$ and $B_{M,A}/B$. A detailed discussion of this equation is given in a later section.

**Figure 1:**
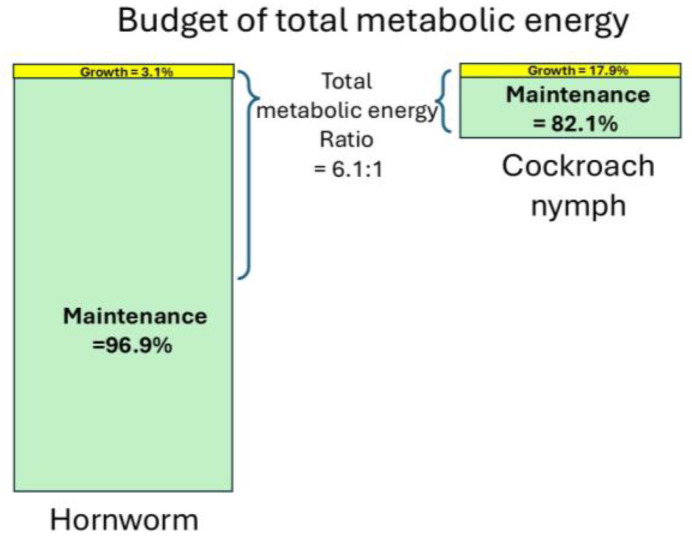
Energy budget of total metabolic energy of hornworms and cockroach nymphs.

Our lab has found [13] that the metabolic rate of painted lady caterpillars (*Vanessa cardui*) is roughly 6.1-fold that of Turkestan cockroach nymphs (*Blatta lateralis*), averaged over the dry body mass range 0.002~0.15 g, and the caterpillars’ growth rate (dry body mass gain per day) is about 27-fold that of the cockroach nymphs (in terms of dry mass). Surprisingly, the fast-growing butterfly caterpillar allocates only 3.1% of its metabolic energy to biosynthesis, only 1/6 of that of the relatively slow-growing cockroach nymphs (17.9%) (Figure 1). Considering the difference in total metabolic rate and the fraction for biosynthesis, the total metabolic energy allocated to biosynthesis is roughly the same in both species (6.1 × 1/6 = 1.06, the total areas of the yellow rectangles in Figure 1). The energy allocated to somatic maintenance and activity in caterpillars is 7-fold that of cockroaches (the average of the ratio *B*_M,A_ = 2982.4 *M*^0.804^/*B*_M,A_ = 1261.0 *M*^1.165^, *M* varies from 0.002 to 0.15 g).

Conventionally, ecophysiologists have focused on how energy allocations tradeoffs between growth and somatic maintenance affect aging and lifespan [8,14,15,16,17,18,19,20,21,22,23,24,25,26,27,28,29,30,31,32]. However, we argue that this explanation may not be enough to depict the whole picture of species’ life history tradeoffs. Hornworms allocate a larger fraction of metabolic energy to somatic maintenance (7-fold) than cockroaches, but they do *not* have better somatic maintenance, as discussed in the following sections, indicating amount of energy allocation may not be the only fact that determines the quality of maintenance [13,33]. What are the life history reasons for such a difference in energy allocation to growth and somatic maintenance between these species? We will discuss this issue in detail in Section 3 with more evidence on cellular resistance to stress.

### 2.2. Energy Budgets Under Food Restriction

Animals may change their energy budget to cope with a low-food-availability situation. The assimilated energy from food is partitioned between metabolic energy and the energy deposited in bio-tissue (growth). Note that here, the energy deposited in bio-tissue is the *direct* energy cost of growth, which is different than the term of $E_{m}G$, which is the indirect energy cost of growth and, as a part of metabolic energy, is dissipated as heat. Since hornworms must reach a threshold of body mass to pupate in a short period, when food availability is low, they would prioritize growth at the cost of maintenance [7]. But cockroaches take a different strategy. Due to their relatively long growth period, they can resume growth after the low-food-supply period, and therefore do not have to maintain a fast growth. So, they may prioritize metabolism during growth. A recent study agrees with these predictions.

**Figure 2:**
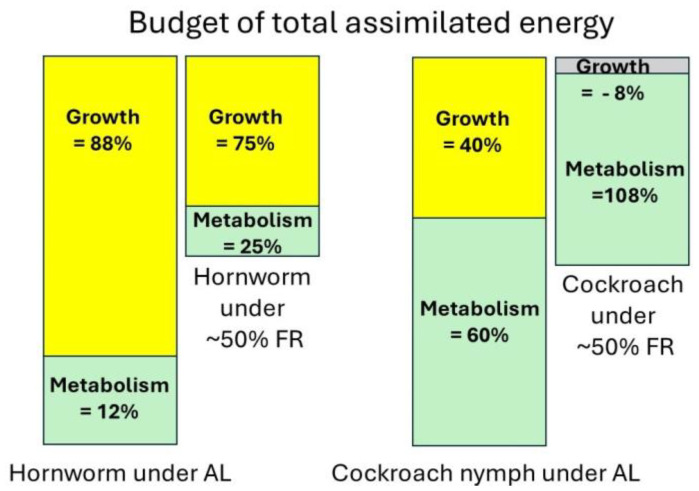
Energy budget of total assimilated energy of hornworms and cockroach nymphs.

We have found recently [34] that hornworms (*Manduca sexta*) allocated ~12% of the assimilated energy to metabolism and ~88%  to growth under ad libitum (AL) conditions. Under food restriction (FR), the fraction allocated to metabolism increased to ~25%, and that to growth is reduced to ~75% (Figure 2). Under AL conditions, orange head cockroach nymphs (*Eublaberus posticus*) allocate ~60% of the assimilated energy to metabolism and ~40%  to growth (Figure 2). But under a similar level of food restriction (~50% of AL) to hornworms, cockroach nymphs spend ~108% of assimilated energy to metabolism, and ~−8% to growth (Figure 2). This means that under FR, cockroach nymphs lost body weight and greatly reduced the energy allocated to growth (~−8%); the available energy that would have been allocated to growth was channeled to metabolism under FR. Under a similar condition, the fraction of assimilated energy allocated to growth was only slightly reduced in hornworms, in agreement with the life history prediction.

Note, in Section 2.1, what we discussed is the partition of metabolic energy between energy cost of biosynthesis (*indirect* cost of growth) and somatic maintenance, both of which are dissipated as heat, whereas in this section, we discuss the partition of assimilated energy between respiration and the *direct* cost of growth, whereby the latter is deposited in biomass, not dissipated.

### 2.3. The Differences in Energetic Costs of Biosynthesis

In Equation (1), we introduced a parameter, the metabolic cost of growth, *E_m_*. As said above, it is the indirect cost of growth and dissipated as heat. *E_m_* is the metabolic work required to synthesize one unit of biomass (in the unit of energy/mass), including the cost of assembling the monomers to polymers, folding them, transporting to the required location, checking the newly synthesized materials for errors, and refolding or degrading misfolded new proteins [9,12,35,36]. Equation (1) indicates that for a given fraction of metabolic energy allocated to growth, *E_m_G*, the higher *E_m_* is (the more expensive biosynthesis is), the slower the growth is.

Since *E_m_* has been considered a fundamental biochemical property of cells [37,38], all the theoretical studies, as far as we are concerned, such as the metabolic theory of ecology [12,39] and the dynamic energy budget theory [40,41], have treated it as a constant across species. Previous empirical studies on endothermic and ectothermic species have found *E_m_* varying within relatively narrow ranges inter-specifically (e.g., [11,35,42,43]). Some studies on insects (e.g., [44,45]) assumed the energy allocated to maintenance and activity is negligible, and reduced Equation (1) to *B* = *E_m_G*, and therefore overestimated the value of *E_m_* of insects.

In a recent study [13], we estimated the *E_m_* values of painted lady caterpillars, hornworms, and Turkestan cockroach nymphs. We found a surprising 20-fold variation in *E_m_* among these species, with 336 Joules/gram of dry body mass (gdbm), 1304 J/gdbm, and 6905 J/gdbm for butterfly caterpillars, hornworms, and cockroach nymphs, respectively. The variation is not caused by the diet and activity levels of the animals [13].

Going beyond insect species, in one of our recent studies [9], using data from 139 mammalian species collected by [46] and the AnAge database [47], we employed a simple mathematical model and estimated that the average value of *E_m_* is 5205 ± 5869 Joules/gram (N = 139) with a 100-fold variation and a coefficient of variation 113%, independently of body mass. The detail is available in [9].

**Figure 3:**
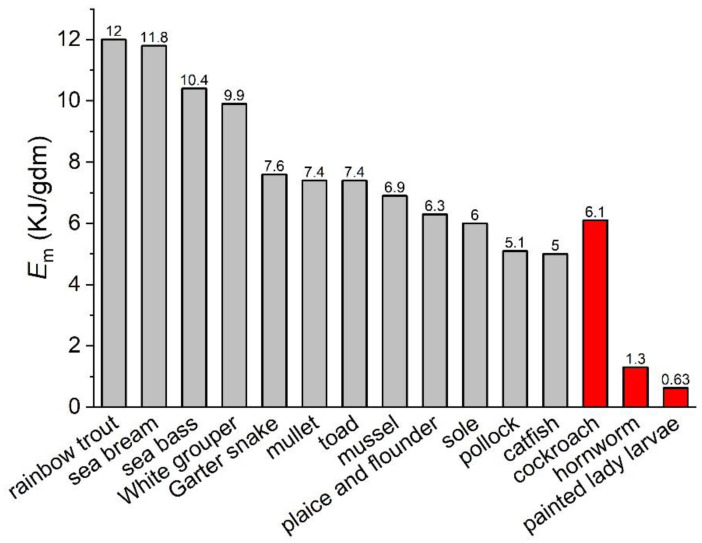
Values of *E_m_* of a few ectothermic species. The red bars represent the species discussed in this paper. (Data from [11,13,43,45,48,49,50,51,52,53].)

Figure 3 shows the values of *E_m_* from some ectothermic species. They range from 5.0 to 12.0 KJ/gdm with an average of 8.0 ± 2.5 KJ/gdm. The *E_m_* of cockroaches, 6.9 KJ/gdm, is in this range, and is comparable to most of the ectothermic species. However, the values of the butterfly caterpillar and hornworm, 0.34 and 1.3 KJ/gdm, are 10 and 5 times lower than the average, respectively, and also much lower than that of catfish (5.0 KJ/gdm), the lowest in Figure 3.

What causes the high variation in *E_m_*?

Proximately, *E_m_* is in part determined by the cell’s tolerance to errors in the syntheses of building materials. Take protein as an example. Protein synthesis and nascent protein folding process is imperfect. The frequency of protein translation errors is estimated to vary from 10^−3^ to 10^−6^ among organisms [54,55,56,57]. It was estimated that 20–30% of nascent polypeptides are rapidly degraded after being synthesized [58,59]. Different species have different degrees of error tolerance and protein quality control. For example, the mouse proteome would have 2- to 10-fold higher levels of proteins with mis-incorporated amino acids relative to the naked mole-rats [60]. Thus, in the species with higher values of *E_m_* (low tolerance to errors), the protein quality control mechanisms need to constantly proofread for mistakes, quickly unfold and refold newly synthesized proteins with errors via the chaperon activities, and/or degrade and resynthesize via the proteasomal and/or autophagy system [61,62,63]. Not only do these processes themselves cost energy [64,65,66,67], but they also slow down the overall biomass growth rate, so that for a unit of net biomass synthesized, a species with a low tolerance to synthetic mistakes would spend more energy (higher *E_m_*) than a species with a high tolerance (lower *E_m_*). On the other hand, compared to species that have a higher tolerance to errors (lower *E_m_*), the “pickier” species that spend more energy on biosynthesis (higher *E_m_*) are more resistant to stress, especially under oxidative stress, due to a better ability of maintaining protein homeostasis [68,69].

We postulate that the ultimate reason for the variation comes from the difference in the life histories between *Lepidoptera* and *Blattodea*. During the metamorphosis of *Lepidoptera* species, only imaginal discs and the tracheal system remain [70,71,72], and a large portion of tissues are disintegrated and remodeled, serving as an energy storage for future reproduction [73,74]. Considering the tissue disintegration during metamorphosis and the requirement of fast growth to reach a threshold body mass in a short period, a high cost of synthesizing tissues with high cellular quality would be economically inefficient and would be selected against. In contrast, the Turkestan cockroach has a long developmental period of 18–40 weeks, and a long adult lifespan of 33–55 weeks [4]. These traits are comparable to other ectothermic species in Figure 1, so they have similar *E_m_* values that are higher than *Lepidoptera* species.

Due to the proximate reasons for the variations in *E_m_*, we predicted that species with different life histories have different cellular and molecular qualities and resistance to stress. Moreover, since protein homeostasis has been repeatedly suggested to play a key role in the aging process [57,58,75,76,77,78,79,80,81], the energy allocation to biosynthesis probably also influences the lifespan of species. In the next two sections, we will discuss these issues.

## 3. Differences in Cellular Quality and Resistance to Stress

### 3.1. Proteasomal Activities

Enhanced nascent protein quality control mechanisms, on one hand, cost a considerable amount of energy [64,65,66,67], on the other hand, will enhance protein homeostasis [57,58,68,69,75,76,77,78,79,80,81]. One of the branches for protein quality control is proteasomal activity. Nascent polypeptides are sensitive to proteotoxic stress, and therefore have high chances of misfolding and aggregation, so they are selectively degraded by the ubiquitin–proteasome machinery (UPS) [76,82,83,84,85,86]. Since the proteasomal activities cost considerable amount of energy [64,76,82,87,88], cells from the species with lower tolerance to the errors in nascent protein will have higher frequent refolding and degrading activities, and therefore spend more energy on depositing one unit of biomass (higher *E_m_*).

In agreement with these predictions, we have found [33] that proteasome activities in the thorax tissues from painted lady caterpillars is 3-fold (N = 26, *p* < 0.001) and 3.5-fold (N = 29, *p* < 0.001) lower than that of Turkistan cockroach nymphs (N = 24 and 25) reared at two temperatures, 22 °C and 30 °C, respectively.

### 3.2. The Ratio of RNA and Organismal Growth Rate

Species with higher *E_m_* values (the “picky” species) have a low error tolerance to the aberrant nascent proteins, so degree of degradation of nascent protein is large. This means their cells allocate a large percentage of protein output from a ribosome to replacing the newly synthesized proteins that have errors and degraded. Thus, these species should have a higher ratio of RNA concentration to organismal growth rate (RNA/GR) [89]. Such variation has been observed in microbes and metazoans [89,90,91,92,93]. Our results showed [33] that the RNA/growth ratio of cockroach (276.4 ± 318.6/(g/day), N = 30) is significantly higher (F = 22.061 and *p*< 0.0001) than that of caterpillars (3.246 ± 1.632/(g/day), N = 30).

### 3.3. Cellular Resistance to Stress

Under stress, cells will first initiate a series of defending and repairing mechanisms, such as the heat shock protein activities that assist refolding and degrading proteins [62,94,95]. If the stressful stimuli continue, cells will activate death signaling pathways [96]. We predict that proteins and cells harvested from species with higher *E_m_* have better resistance to stresses, at least partially because they allocate more energy to the protein quality control mechanisms.

We have [33] used flow cytometry to assay the cell viability and apoptosis in painted lady caterpillar and cockroach nymph midguts under oxidative stress induced by tert-butyl hydroperoxide (t-BHP) at eight concentrations, 0, 3, 6,12,15, 50, 100, and 200 mM. Simultaneous staining of cells with 7-aminoactinomycin and Annexin V–FITC allows the discrimination of intact cells, early apoptotic cells, and late apoptotic and dead cells. Except for the two highest concentrations of t-BHP (100 and 200 mM), which caused the extremely low cell viability of both species, at other concentrations, caterpillars have significantly higher percentages of the sum of apoptotic and dead cells than cockroaches. Using Annexin V and 7AAD double staining, we obtained the percentages of apoptotic cells. The difference between the t-BHP-induced apoptosis in painted lady butterfly caterpillars and cockroach cells was significant only at two medium concentrations (12 and 15 mM), indicating that cockroach cells have a higher resistance to apoptosis than caterpillar cells.

Based on the evidence of the differences in energy budget and cellular resistance to stress between these species, we argue that the capability of maintaining homeostasis not only depends on the amount of energy allocated to maintenance, but also depends on the quality of the tissue, such as the endurance to oxidative stress, and that the tissue quality is at least partially due to the energetic investments in biosynthesis. Differently but not in opposition to the suggestion by the conventional life history theory that allocating more energy to somatic maintenance may retard aging, we postulate a new form of tradeoff; i.e., materials that are cheap to synthesize deteriorate faster, and allocating more energy to biosynthesis enhances the somatic maintenance.

## 4. Future Research

In previous studies, we have only investigated moth and butterfly caterpillars and the nymphs of two cockroach species. To fully understand how species life history affects the physiological traits, more species with different life histories, especially different growth rates, need to be studied. In particular, more efforts need to be made to investigate the values of *E_m_* in insects, because of its significance in physiology and ecology [11,35,41,52,97,98,99]. The common method of measuring *E_m_* requires the entire growth curve and metabolic rates at different ages. Assuming the metabolic energy for maintenance and activity (the term *B*_M,A_ in Equation (1)) scales with body mass *M*(*t*) (a function of age) as $B_{M,A}(t)=a{M(t)}^{b}$, where *a* is a normalization coefficient and *b* is a scaling power. Here, *b* is not the conventional scaling power of metabolic rate, which usually varies between 0.66 and close to 1 [100,101,102]. Instead, it shows how maintenance term scales with body mass. In a simplified version of this equation, West and his colleagues assumed that *b* is equal to one [12]. This assumption is not needed here, as *b* will be empirically determined by the data. Equation (1) thus becomes $B(t)=E_{m}G(=\frac{dM\left(t\right)}{dt})+a{M(t)}^{b}$. Once the growth rate, *G*(*t*) =dM(t)/dt, metabolic rate, *B*(*t*), and body mass, *M*(*t*), all as functions of age, are empirically obtained, one will obtain three columns of data points (as functions of time), *B*(*t*), *G*(*t*), and *M*(*t*). The nonlinear regression of *B*(*t*) on *G*(*t*) and *M*(*t*) will yield the values of *E_m_* as well as parameters *a* and *b* [52,103]. In general, the more data points taken during ontogenetic growth, the more accurate the results of nonlinear regression will be. Note that here, one does not need to assume how metabolic rate scales with body mass over ontogeny, although the scaling power can be obtained by measuring *B*(*t*) and *M*(*t*). What are needed are just values of the metabolic rate at different time points during ontogeny. This method has been commonly applied (e.g., see [43,52,103]). More complex ontogenetic growth models, such as dynamic energy budget (DEB) [41] may capture more details of the ontogenetic dynamics, but such models require measurement of enormous amounts of variables and parameters during growth, which rendered them not as practical as the simple growth model. Moreover, perhaps due to this reason, as far as we are concerned, all the studies using DEB assume a constant *E_m_* across species and during growth.

If the variation in *E_m_* of insects is confirmed, the next step is to investigate how nascent protein quality control mechanisms vary with life histories. Here, we emphasize two important branches in the nascent protein quality control mechanisms that need to be investigated. The first branch is related to translation fidelity. The rate of mis-incorporation during translation is one in every 5000–10,000 amino acids in eukaryotic cells [66,104,105,106]. Improving the fidelity costs energy, because ribosomes need to search for the correct aminoacyl-tRNA based on the codon-anticodon pairing, which involves elongation factors that bind GTP [59,104]. The hydrolysis of GTP to GDP provides the energy necessary for the correct tRNA to be accepted into the ribosome [107,108]. It has been shown that the variation in the energy expenditure in this process comes from the effect of competition by cognate tRNAs for the mutant codons [104].

Second, the newly translated polypeptides need to be correctly folded by the molecular chaperons, which also identify the misfolded proteins, promote refolding, and assist degradation [62,64,76,109,110,111]. It also has been shown that nascent polypeptides are sensitive to proteotoxic stress, and therefore have high chances of misfolding and aggregation, so they are selectively degraded by the ubiquitin–proteasome machinery (UPS) [76,82,83,84,85,86].

Since the chaperon and proteasomal activities cost considerable amount of energy [64,76,82,87,88], cells from the species with lower tolerance to the errors in nascent protein will have higher frequent refolding and degrading activities, and therefore spend more energy on depositing one unit of biomass (higher *E_m_*).

A more important question is how *E_m_* would affect species lifespan. As mentioned above, protein homeostasis has been repeatedly suggested to play a key role in the aging process [57,58,75,76,77,78,79,80,81]. It is has been recognized that loss of proteostasis is one of the nine major hallmarks of aging [80,81]. As aging progresses, translation slows down and irreparably damaged proteins accumulate, which in turn hinders the chaperones’ activities of folding the healthy proteins [112]. Enhancing protein quality control mechanisms has been proven to increase the lifespans of organisms. For example, Ke et al. [54] have found strong positive correlation between translation fidelity and maximum lifespan across 17 rodent species with diverse lifespans; Martinez-Miguel et al. have shown [57] that introducing a mutant (RPS23 K60R) that improves the accuracy of protein synthesis increases the lifespans of yeast, *C*. *elegans*, and fly and the ability of heat resistance. Some drugs, such as rapamycin, torin1, and trametinib, increase translation accuracy [57], and meanwhile, they increase lifespan in all model organisms studied [113].

This evidence suggests that species with a higher *E_m_* may also have a longer lifespan if body mass and metabolic rate are controlled. Our recent study has proven it true in mammalian species [9]. Using data from 133 mammalian species, we found that after correcting the mass-specific metabolic rate, the maximum lifespan is positively correlated with *E_m_*.

However, it may not be true in insects, especially holometabolous species. This is because during complete metamorphosis, most of the body tissues are degraded and re-built, so there is a discontinuity between the quality of cells built during the larval stage and that during adulthood. For example, Campero et al. have shown that metamorphosis offsets the link between larval stress by food restriction and pesticide, adult asymmetry, and individual quality in damselflies (*Calopteryx virgo*) [114]. So, for moth species, how larval *E_m_* affects adult insect lifespan is unclear. We call for future research on the link between the energy cost of biosynthesis during growth and adult lifespan in insects.

The purpose of this paper is to compare the differences in the energy allocation strategies between two taxa and the consequences on their cellular qualities and aging processes. Needless to say, there are alternative pathways for cellular repair, such that the energetic cost of biosynthesis may not directly relate to resistance to cellular stress, such as telomere maintenance [115], enhancement of intercellular communication [116], nutrient sensing maintenance [117], etc. A deeper discussion on these pathways is beyond the scope of this paper. The details of how these pathways are related to animal energy budget is yet to be investigated.

## Data Availability

No new data were created.
